# Targeting Id1 reduces proliferation and invasion in aggressive human salivary gland cancer cells

**DOI:** 10.1186/1471-2407-13-141

**Published:** 2013-03-22

**Authors:** Tomoki Sumida, Ryuichi Murase, Akiko Onishi-Ishikawa, Sean D McAllister, Hiroyuki Hamakawa, Pierre-Yves Desprez

**Affiliations:** 1 Department of Oral and Maxillofacial Surgery, Ehime University School of Medicine, 454 Shitsukawa, Toon-City, Ehime, 791-0295, Japan; 2 California Pacific Medical Center, Cancer Research Institute, San Francisco, CA, 94107, USA

**Keywords:** Inhibitor of differentiation, Id2, Id3, Promoter assay, ACCM cells

## Abstract

**Background:**

Salivary gland cancer (SGC) is one of the common malignancies of the head and neck area. It develops in the minor and major salivary glands and sometimes metastasizes to other organs, particularly to the lungs. Inhibitors of differentiation (Id) proteins are negative regulators of basic helix-loop-helix transcription factors that control malignant cell behavior and tumor aggressiveness in many tissues. In this study, our goal was to determine the potential role of Id proteins, particularly Id1, during human SGC cell progression.

**Methods:**

We first determined the expression levels of Id1 and Id2 in four SGC cell lines: two adenocarcinoma of the salivary gland (HSG and HSY) and two adenoid cystic carcinoma (ACC2 and ACCM) cell lines. We then used constructs that expressed antisense cDNAs to Id1 or Id2 to knockdown the expression of these proteins in cell lines where they were highly expressed, and determined the effects of the knockdown on cell proliferation, migration and invasion.

**Results:**

Id1 mRNA and protein were detectable in all cell lines, and expression of Id2 was variable, from absent to high. The ACC2 and ACCM cell lines expressed both Id1 and Id2, but Id1 was expressed at a higher level in the more aggressive ACCM cell line in comparison toACC2 cells as confirmed by *Id1* promoter-reporter assays. We therefore focused on the ACCM cells for the remainder of the study. We found that proliferation and invasiveness of ACCM cells were strongly reduced after Id1 knockdown whereas Id2 suppression had only a slight effect. Results of scratch and colony formation assays also confirmed that ACCM cell aggressiveness was significantly reduced upon Id1 knockdown. Finally, this knockdown resulted in reduced c-myc and enhanced cyclin-dependent kinase inhibitor p21 expression.

**Conclusions:**

These results demonstrate that Id1 plays an important role in the control of human SGC cell aggressiveness and suggest a potential role as a marker of diagnosis, prognosis and progression of SGCs. Id1 suppression could represent a novel and effective approach for the treatment of salivary gland cancer.

## Background

The development of aggressive cancers is a multistep process involving many genetic and epigenetic alterations. Identifying these alterations is essential to understanding the mechanisms of cancer progression, and will enable the development of more effective methods for diagnosis and treatment. Human salivary gland cancer (SGC) is a typically slow-growing neoplasm of the secretory glands, most common in the minor and major salivary gland [1,2]. However, SGCs also include highly aggressive tumors that invade the adjacent tissues and metastasize to distant organs at an early stage [1,3]. Some of the most common malignant SGCs correspond to adenoid cystic carcinomas (ACC) and the survival rates for this type of cancer at 10 and 20 years are extremely poor [3-5]. Recurrent cases of ACC are particularly difficult to manage because of the ineffectiveness of radio- and chemotherapy as well as the cosmetic and anatomic limitations in performing wide surgical resection [6,7]. Therefore, a new treatment modality for SGCs is urgently needed.

Recently, high expression levels of inhibitor of differentiation (*Id*) genes have been observed in cell lines derived from a variety of tumors and tumor tissues, suggesting that Id proteins have been implicated in cancers originating from many organs [8]. Id proteins are a class of helix-loop-helix (HLH) transcriptional regulators. Constitutive expression of these proteins inhibits the differentiation of various cell types through their interaction with basic helix-loop-helix (bHLH) proteins [9,10]. bHLH transcription factors are key regulators of lineage- and tissue-specific gene expression in a number of mammalian and non-mammalian organisms. bHLH proteins act as obligate dimers binding DNA through composite basic domains to regulate the transcription of target genes containing E-boxes (CANNTG) in their promoters. Id proteins dimerize with bHLH proteins, but because Id proteins lack basic domains, Id-bHLH heterodimers fail to bind DNA. Therefore, Id proteins are dominant negative regulators of bHLH function [9,11].

To date, four members of the *Id* gene family have been described (*Id1*-*Id4*) [12,13]. They are located on different chromosomes and have different expression patterns and functions. We hypothesized that Id proteins may be involved in the regulation of SGC cell proliferation and invasiveness. As a first step, we chose to focus our investigations on Id1 and Id2. Id4, however, also has a strong association with some types of tumors [14,15] and Id3 displays similar expression pattern as Id1 [16]. We found that both Id1 and Id2 were highly expressed in ACC2 and particularly ACCM cells, an aggressive sub-clone of ACC2. However, only Id1 knockdown but not Id2 knockdown triggered a significant reduction in the proliferative and invasive phenotype of SGC cells, suggesting an important role of Id1 in the regulation of SGC cell aggressiveness.

## Methods

### Cell culture

Four cell lines, all derived from human SGCs, were used for the studies. HSG cells were established from an adenocarcinoma of the submandibular gland [17], HSY cells were also established from an adenocarcinoma [18], ACC2 cells were established from an adenoid cystic carcinoma, and ACCM cells were a subpopulation of ACC2 with a highly aggressive phenotype [19]. The HSG and HSY cell lines were a generous gift from Prof. Sato (Tokushima University, Tokushima, Japan). The ACC2 and ACCM cell lines were purchased from the Cell Bank of Type Culture Collection of the Chinese Academy of Science (CBTCCCAS, Shanghai, China). All cell lines were cultured in RPMI 1640 (University of California, San Francisco, CA) supplemented with 10% FBS and 5 μg/ml insulin (Sigma) at 37°C in the presence of 5% CO_2_. For the serum-free conditions, FBS was omitted from the medium.

### Western blot analysis

Cells were lysed in 2X Laemmli buffer [20] and stored at −70°C. The protein concentration was determined using the DC protein assay kit (Bio-Rad). Samples (20–30 μg of total protein) were separated by SDS-PAGE and transferred to PVDF membranes (Hybond P; Amersham) using standard methods [20]. Membranes were blocked for 1 h at room temperature with TBST (20 mM Tris, 137 mM NaCl, 3.8 mM HCl, and 0.1% Tween-20) containing 5% nonfat milk, and blots were probed with anti-Id1, anti-Id2, anti-Id3, anti-p21, and anti-c-myc (Santa Cruz Biotechnology), or anti-actin (Chemicon) antibodies for 1 h. Membranes were washed and incubated with a secondary antibody (either goat anti-rabbit or anti-mouse IgG-horseradish peroxidase; Santa Cruz Biotechnology), washed again, and developed for enhanced chemiluminescence using the Amersham ECL-Plus kit according to the supplier’s instructions.

### RNA extraction and northern blot analysis

RNA was isolated and purified as described by Chomczynski and Sacchi [21], and 15 μg of total RNA was separated by electrophoresis using formaldehyde-agarose gels, and transferred to a nylon membrane (Hybond N; Amersham). The membrane was hybridized using either a ^32^P-labeled human Id1 [22] or Id2 [23] cDNA probe, washed, and exposed to a XAR-5 film for autoradiography, as described previously [24]. Ribosomal 28S and 18S RNA were used as loading controls and to determine RNA integrity.

### Id1 promoter-reporter assays

We used a 2.2-kb fragment corresponding to the 5-upstream region of the human *Id1* gene driving a luciferase gene (*Id1*-sbsluc) in the PGL-3 vector (Promega) as previously described [25]. Cells were plated in 6-well dishes at a density of 3x10^5^ cells per well in RPMI 1640 medium supplemented with 10% FBS and 5 μg/ml insulin. After 24 h, cells were co-transfected with 6 μg of luciferase reporter plasmids and 2 μg of pCMVβ (Clontech) using SuperFect transfection reagent (Qiagen). Vector pCMVβ, containing bacterial β-galactosidase driven by the constitutive CMV promoter, served as a control for variation in transfection efficiency. Three hours after transfection, cells were rinsed twice with serum-free medium, cultured in RPMI 1640 medium with 10% FBS and 5 μg/ml insulin for 48 h, scraped into 1 ml of PBS and collected by centrifugation (13,000 rpm for 10 min). Cell pellets were re-suspended in 80 μl of reporter lysis buffer (Promega) and incubated for 10 min at room temperature. After centrifugation, supernatants were collected and used for luciferase and β-galactosidase assays using the Luciferase Assay System (Promega), β-Galactosidase Assay Kit (Clontech), and a 2010 luminometer (Pharmingen). Luciferase activities were normalized to β-galactosidase activities.

### The pBabe-Id1 retroviral vector and virus production

The full-length human *Id1* cDNA was excised from CMV-*Id1* and cloned into pBabe-puro [25], a gift from Dr. Hartmut Land (ICRF, London, UK). Clones in which the *Id1* cDNA was inserted in the antisense orientation (pBabe-*Id1*AS) were selected for use. The full-length human *Id2* cDNA, a gift from Dr. Eiji Hara (Manchester, UK), was also cloned into a pBabe vector in the antisense orientation (pBabe-*Id2*AS). Either pBabe-*Id1*AS or pBabe-*Id2*AS was transfected into the TSA54 packaging cell line (Cell Genesis) using calcium phosphate [26]. Twenty-four hours after transfection, the culture medium (containing infectious virus) was harvested twice at 24 h intervals and frozen at −80°C. Viral titers were determined using an assay to detect reverse-transcriptase activity.

### Retroviral infection

Approximately 8 RT units of pBabe-ctl (empty plasmid), pBabe-*Id1*AS or pBabe-*Id2*AS was mixed with 5 ml of a medium containing 4 μg/ml polybrene and added to cells in 100-mm dishes. Cells expressing the retroviral genes were selected using puromycin. The antibiotic resulted in the death of all mock-infected cells within 3 days, and the surviving cells infected with pBabe-ctl, pBabe-*Id1*AS, or pBabe-*Id2*AS were harvested.

### [^3^H]Thymidine incorporation assay

Cells were cultured on coverslips in 1% serum, [^3^H]thymidine (μCi/ml; 60–80 Ci/mmol; Amersham) was included during the final 16 h of culture, and cells were fixed with a 1:1 methanol and acetone (vol/vol) solution. Cell nuclei were stained with DAPI, diluted 1:10,000 in PBS. [^3^H]Thymidine-labeling was developed as previously described [27]. The percentage of labeled nuclei was calculated by comparing the number of [^3^H]thymidine-labeled nuclei with the number of DAPI-stained nuclei in a given field using light and fluorescence microscopy.

### Boyden chamber invasion assay

Invasion assays were performed in modified Boyden chambers using 8 μm pore filter inserts in 24-well plates (Collaborative Research). The filters were coated with 10–12 μl of ice-cold Matrigel (Collaborative Research). Cells (approximately 80,000/well) were added to the upper chamber in 200 μl of the appropriate medium containing 0.1% BSA, and the lower chamber was filled with 300 μl of NIH-3T3 cell-conditioned medium. Assays were performed in triplicate or quadruplicate, and the results were averaged. After incubating for 20 h, cells were fixed with 2.5% glutaraldehyde in PBS and stained with 0.5% toluidine blue in 2% Na_2_CO_3_. The cells remaining in Matrigel or those attached to the upper side of the filter were removed with cotton tips. Cells on the lower side of the filter were counted under light microscopy.

### Cell migration assay

Cells were seeded in 6-well plates at a density of 1.0 × 10^5^ cells per well. The following day, a scratch wound was generated by scratching the bottom of the wells with a pipette tip. Wells were rinsed with media to remove the detached cells, and replaced with media containing 10% serum. Cultures were then maintained for 24 h. Photographic images of each well were taken immediately following wound generation and again after 24 h. NIH Image (National Institutes of Health, Bethesda, MD) was used to measure the areas not covered by the migrating cells at each time point. The experiments were performed in triplicate.

### Colony formation assay

Infected ACCM cells were trypsinized, re-suspended as single cells, and plated in 6-well plates (coated with 0.5% soft agar) at a density of approximately 100 cells per well diluted in the upper layer of 0.33% soft agar. After 2 weeks, colonies were fixed with methanol and stained with crystal violet. Colony numbers were counted under the microscope. The number of colonies observed in antisense groups were normalized and presented as a percentage of the average number of colonies in control groups.

### Statistical analyses

Differences between groups were determined using a Student’s *t*-test. P values <0.05 were considered statistically significant. All statistical tests were performed using Statcel2 software (Statcel2, OMS, Tokyo).

## Results

### Id1 and Id2 gene expression in SGC cells

**Figure 1:**
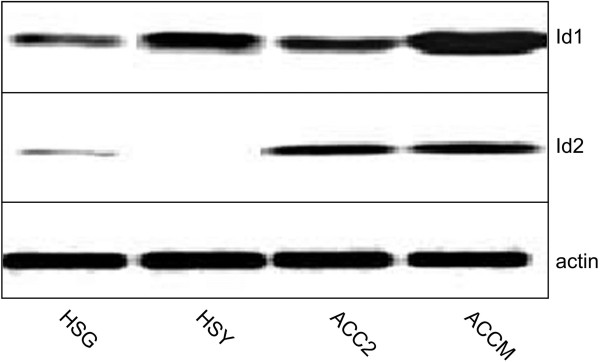
**Id1 and Id2 protein expression in human SGC cell lines.** HSG, HSY, ACC2 and ACCM cells were analyzed for expression of Id1 and Id2 by Western blotting. A loading control was carried out by stripping the blot and re-probing with an anti-actin antibody.

We first used Western blot analysis to compare the expression of Id1 and Id2 among four different SGC cell lines, and to determine which cell line would be the most suitable for knockdown experiments. Whereas all cell lines displayed high levels of Id1 protein expression, Id2 expression was more variable (Figure 1). Western blot analysis demonstrated that ACCM cells, an aggressive sub-clone of ACC2 cells, had the strongest expression of Id1 (and of Id3 as shown in Additional file 1: Figure S1), whereas Id2 was expressed at similar levels in ACC2 and ACCM cells, and almost undetectable in HSG and HSY cells (Figure 1). Therefore, subsequent analysis and experiments were performed using ACC2 and ACCM cells.

**Figure 2:**
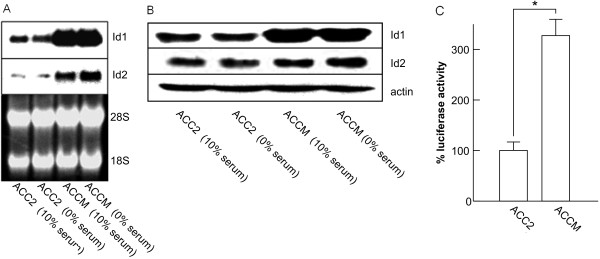
**Id1 and Id2 mRNA and protein expression in SGC cells in the presence or absence of serum.** (**A**) Total RNA isolated from ACC2 and ACCM cells cultured in 10% FBS or in serum-free medium (0%) were analyzed for expression of *Id1* and *Id2* by Northern blotting. To control for RNA quantity and integrity, the ethidium bromide-stained gel is shown in the lower panel. (**B**) Western blot comparing the levels of Id1 and Id2 protein expression in cells cultured under the same conditions as in (**A**) is shown. (**C**) Relative expression of *Id1* in ACC2 and ACCM cells is indicated by the expression of luciferase (a reporter driven by a human *Id1* promoter). * indicates a significant difference between the two cell lines (p < 0.05, Student’s *t* test).

Further analysis of Id1 expression using Northern blotting demonstrated that *Id1* mRNA expression was indeed much higher in ACCM cells than in ACC2 cells in agreement with the analysis of Id1 protein expression (Figure 2A). We also tested whether *Id1* and *Id2* expression was dependent upon the concentration of serum in the media. We found that serum starvation did not have any significant effect on the expression of *Id1* and *Id2* mRNA (Figure 2A) or protein (Figure 2B) (and of Id3 protein as shown in Additional file 2: Figure S2). Next, we performed *Id1* promoter-reporter assays in ACC2 and ACCM cells. These assays confirmed that higher levels of *Id1* expression were present in the most aggressive ACCM cells compared to less aggressive ACC2 cells (approximately a three-fold difference in luciferase activity) (Figure 2C).

### Effect of Id1 and Id2 knockdown on ACCM cell proliferation and invasion

**Figure 3:**
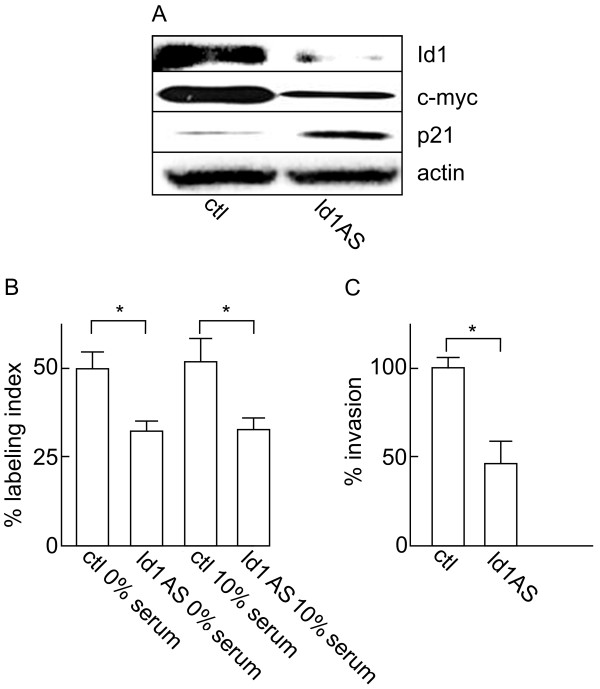
**Effects of Id1 knockdown on proliferation and invasiveness of ACCM cells.** (**A**) Western blot shows the effects of Id1 knockdown (Id1AS) on the expression of the cell proliferation markers c-myc and p21. (**B**) Effect of Id1 knockdown on the proliferation of ACCM cells was determined in 10% serum or serum-free medium (0%) after 48 h. (**C**) Effect of Id1 knockdown on cell invasiveness was determined using Boyden chamber invasion assays. Data represent the average of 6 independent experiments and are presented as percentage of controls (ctl). * indicates significant differences between cell populations (p < 0.05, Student’s *t* test).

In order to investigate the potential role of Id1 and Id2 in the regulation of SGC cell aggressiveness, we used the ACCM cells stably infected with the pBabe-*Id1*AS or pBabe-*Id2*AS constructs. First, Id1 knockdown in the ACCM pBabe-*Id1*AS cells was confirmed by Western blotting (Figure 3A). Using [^3^H]thymidine-incorporation assay, we then determined that Id1 knockdown resulted in a significant reduction in cell proliferation relative to the control group (Figure 3B). Similar to what was observed for Id1 mRNA and protein expression, serum starvation did not have any significant effect on the reduced proliferation rate produced by Id1 knockdown. Id1 knockdown was also associated with modifications of the expression of proliferation markers. Whereas p21 expression was strongly up-regulated, expression of the c-myc oncogene was down-regulated in ACCM pBabe-*Id1*AS cells (Figure 3A). Finally, Id1 knockdown in SGC cells also produced a significant reduction in cell invasion. Using the Boyden chamber invasion assay, we found that the number of invading cells was reduced by approximately 50% in the antisense group when compared to control (Figure 3C).

**Figure 4:**
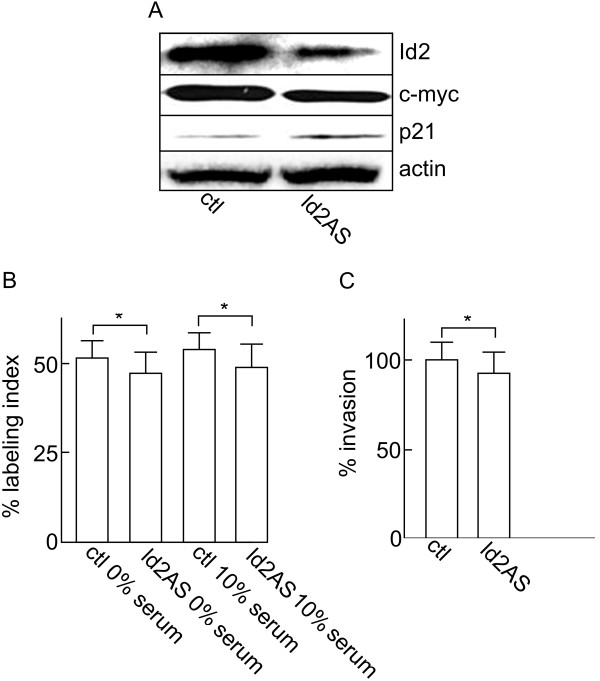
**Effects of Id2 knockdown on proliferation and invasiveness of ACCM cells.** (**A**) Western blot shows the effects of Id2 knockdown (Id2AS) on the expression of the cell proliferation markers c-myc and p21. (**B**) Effect of Id2 knockdown on the proliferation of ACCM cells was determined in 10% serum or serum-free medium (0%) after 48 h. (**C**) Effect of Id2 knockdown on cell invasiveness was determined using Boyden chamber invasion assays. Data represent the average of 6 independent experiments and are presented as percentage of controls (ctl). * indicates significant differences between cell populations (p < 0.05, Student’s *t* test).

In contrast to Id1, successful knockdown of Id2 in ACCM cells (Figure 4A) did not lead to a significant decrease in the proliferation rate or invasion of the cancer cells (Figure 4B and C). In addition, only a minor reduction in c-myc and increase in p21 expression was observed compared to control cells (Figure 4A).

### Id1 but not Id2 regulates cell migration and ability of ACCM cells to form colonies

**Figure 5:**
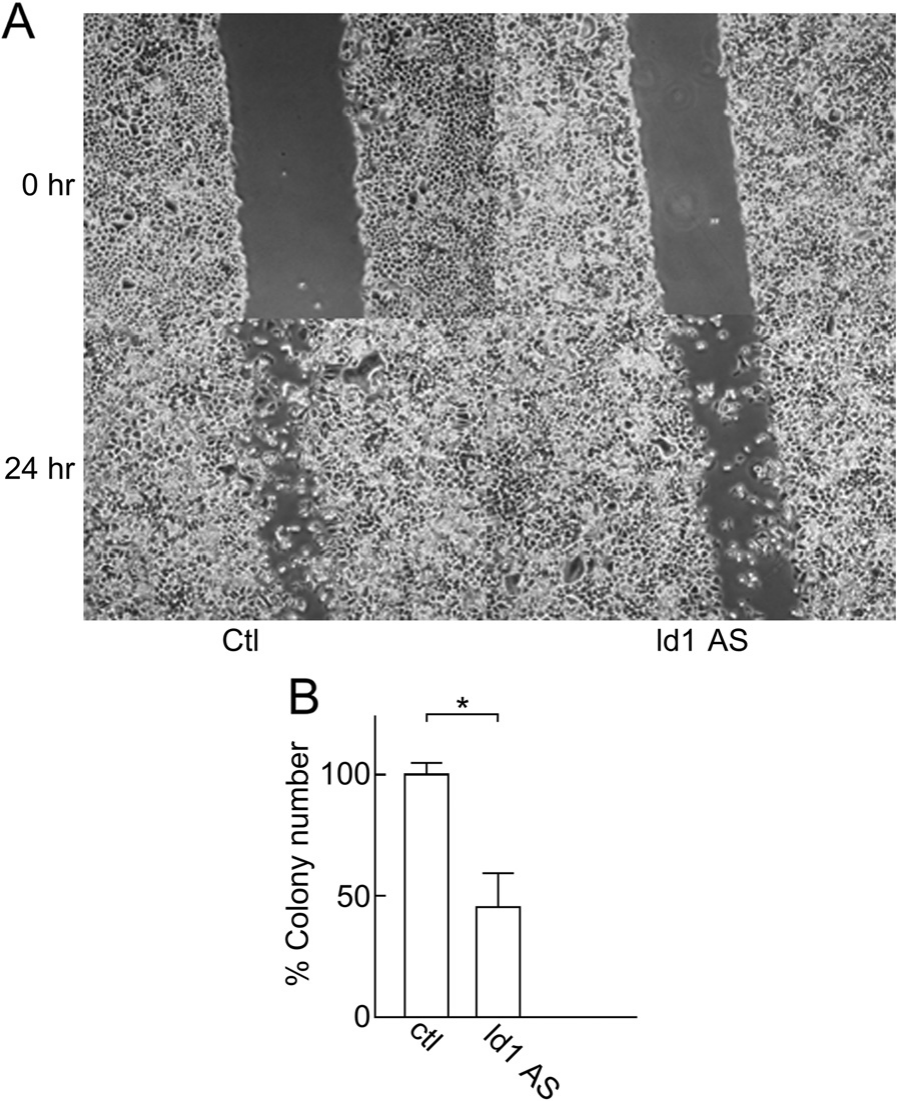
**Effects of Id1 knockdown on ACCM cell migration and colony formation.** (**A**) Images show scratch wounds using pipette tips and compare ACCM-control cells (Ctl) and ACCM-Id1-antisense cells (Id1AS) immediately (0 hr) or 24 hr after scratches were inflected. (**B**) Results of colony formation assays comparing ACCM-control cells (Ctl) and ACCM-Id1-antisense cells (Id1AS) are shown. Bars represent the percentage of number of colonies in Id1AS cells relative to control cells. * indicates a significant difference between cell populations (Student’s *t* test, p < 0.05).

**Figure 6:**
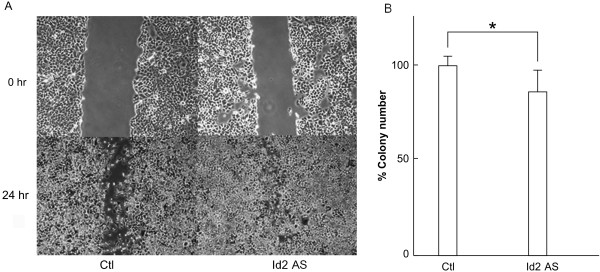
**Effects of Id2 knockdown on ACCM cell migration and colony formation.** (**A**) Images show scratch wounds using pipette tips and compare ACCM-control cells (Ctl) and ACCM-Id2-antisense cells (Id2AS) immediately (0 hr) or 24 hr after scratches were inflected. (**B**) Results of colony formation assays comparing ACCM-control cells (Ctl) and ACCM-Id2-antisense cells (Id2AS) are shown. Bars represent the percentage of number of colonies in Id2AS cells relative to control cells. * indicates a significant difference between cell populations (Student’s *t* test, p < 0.05).

We used scratch and colony formation assays to examine the effects of Id1 and Id2 knockdown on additional aspects of ACCM cell aggressiveness. Id1 knockdown triggered a significant reduction in ACCM cell migration in the scratch assay after 24 h when compared to control cells (Figure 5A; p < 0.05, Student’s *t* test) as well as a significant decrease in the number of colonies when we used the colony formation assay (Figure 5B; p < 0.05, Student’s *t* test). In contrast, Id2 knockdown did not affect cell migration, since almost the entire area of the scratch was filled after 24 h (Figure 6A), and failed to produce a significant decrease in colony formation (Figure 6B). Overall these data are in agreement with our results obtained from the proliferation and invasion assays implicating Id1 as a key factor in SGC cells.

## Discussion

The pathways controlling the proliferation and invasion of aggressive and metastatic ACC are still not well understood, a gap in the field further hindered by the fact that cancer cell lines derived from salivary glands are difficult to obtain. Here, we compared four different SGC cell lines, including the ACCM cell line, a highly metastatic and aggressive variant obtained from the parental ACC2 cells [19]. We found that Id1 mRNA and protein expression was indeed higher in ACCM cells than in ACC2 cells, whereas Id2 protein expression was almost identical in both cell lines. These data suggested that a constitutively high expression of Id1 (independent of the presence of serum) was at least partly responsible for the highly aggressive nature of ACCM cells. Since Id3 was also expressed in these cells and may play a role in the proliferative and/or invasive phenotype of SGCs, it is possible that a double Id1/Id3 knockdown would further increase the reduction of tumor cell aggressiveness.

Overall, the high levels of Id expression detected in ACCM cells suggest that this family of proteins could be responsible for the proliferative, migratory and invasive nature of these cells, and suggest that these transcriptional regulators could serve as diagnostic and/or prognostic factors of SGC. High expression levels of *Id* genes have been observed in cell lines derived from a wide variety of tumors and tumor tissues [28]. Id proteins, especially Id1, are associated with a more aggressive and invasive behavior, as well as with a less differentiated tumor phenotype, and in some kinds of tumors, as markers of cancer diagnosis and progression [28]. Our results demonstrating a different role of Id1 and Id2 in ACCM cells suggest that SGCs differ from other types of cancer in which Id1 and Id2 appear to play a more equivalent role. For example, Id1 and Id2 are both over-expressed in pancreatic cancer cells [29], and are both considered prognostic markers of squamous cell carcinoma metastasis in the esophageal region [30].

Upon Id1 knockdown, c-myc expression was repressed and p21 protein was strongly up-regulated. On the other hand, there was a lack of modulation of c-myc and p21 expression after Id2 knockdown. Therefore, we can speculate that Id1 protein is, at least indirectly, associated with the regulation of cell cycle-associated genes. In prostate cancer cells, silencing Id1 induced the expression of the cell cycle regulatory proteins p16 and p21 and triggered a change in MMP-9 levels [31]. We previously reported that in human aggressive SGC cells [32] and in metastatic breast cancer cells [33], introduction of the progesterone receptor and treatment with progestin was sufficient to reduce Id1 expression, down-regulation of c-myc and MMP-9, up-regulation of p21 and consequently a decrease in the aggressive phenotype of the cells. The down-regulation of proteins such as matrix metallo-proteinases could explain the effects of Id1 knockdown on the reduction of SGC cell invasion.

Moreover, the significant effects of Id1 knockdown, but not of Id2 knockdown, on cell migration (measured using the scratch assay) and on the ability of the cells to grow in an anchorage-independency fashion (measured using the soft agar assay) provide additional support for the key role of Id1 during SGC cell progression.

## Conclusions

The results of this study indicate that Id1 expression is correlated with a highly aggressive phenotype of SGC cells and may be associated with the malignant progression of salivary gland epithelial cells. Knockdown of Id1 decreased the rate of proliferation, growth in soft agar, migration and invasion of ACCM cells, indicating that suppression of Id1 may impact SGC progression in patients. We are now testing the role of Id1 on SGC cell metastasis *in vivo* as the next logical step in understanding the importance of Id1 during SGC progression. In conclusion, we propose that suppression of *Id1* gene expression may represent a novel and effective approach for the treatment of SGC.

## Abbreviations

ACC: Adenoid cystic carcinomas; bHLH: Basic helix-loop-helix; HLH: Helix-Loop-Helix; Id: Inhibitor of differentiation; SGC: Salivary gland cancer.

## Competing interests

The authors declare that they have no competing interests.

## Authors’ contributions

TS designed the study and drafted the manuscript; RM and AOI performed the experiments; SDM, HH and PYD supervised the experiments and edited the manuscript. All authors read and approved the final manuscript.

## Pre-publication history

The pre-publication history for this paper can be accessed here:

http://www.biomedcentral.com/1471-2407/13/141/prepub

## Supplementary Material

Additional file 1: Figure S1 Id3 protein expression in human SGC cell lines. HSG, HSY, ACC2 and ACCM cells were analyzed for expression of Id3 by Western blotting. Loading control was carried out by stripping the blot and re-probing with an anti-actin antibody. Click here for file

Additional file 2: Figure S2 Id3 protein expression in SGC cells in the presence or absence of serum. Western blot comparing the levels of Id3 protein expression in ACC2 and ACCM cells cultured in 10% FBS or in serum-free medium (0%) is shown. Click here for file
