# Supplementary figures and images for: Targeting Id1 reduces proliferation and invasion in aggressive human salivary gland cancer cells
Source: BMC Cancer. 2013 Mar 22;13:141. doi: 10.1186/1471-2407-13-141 (PMC3639030; doi:10.1186/1471-2407-13-141)

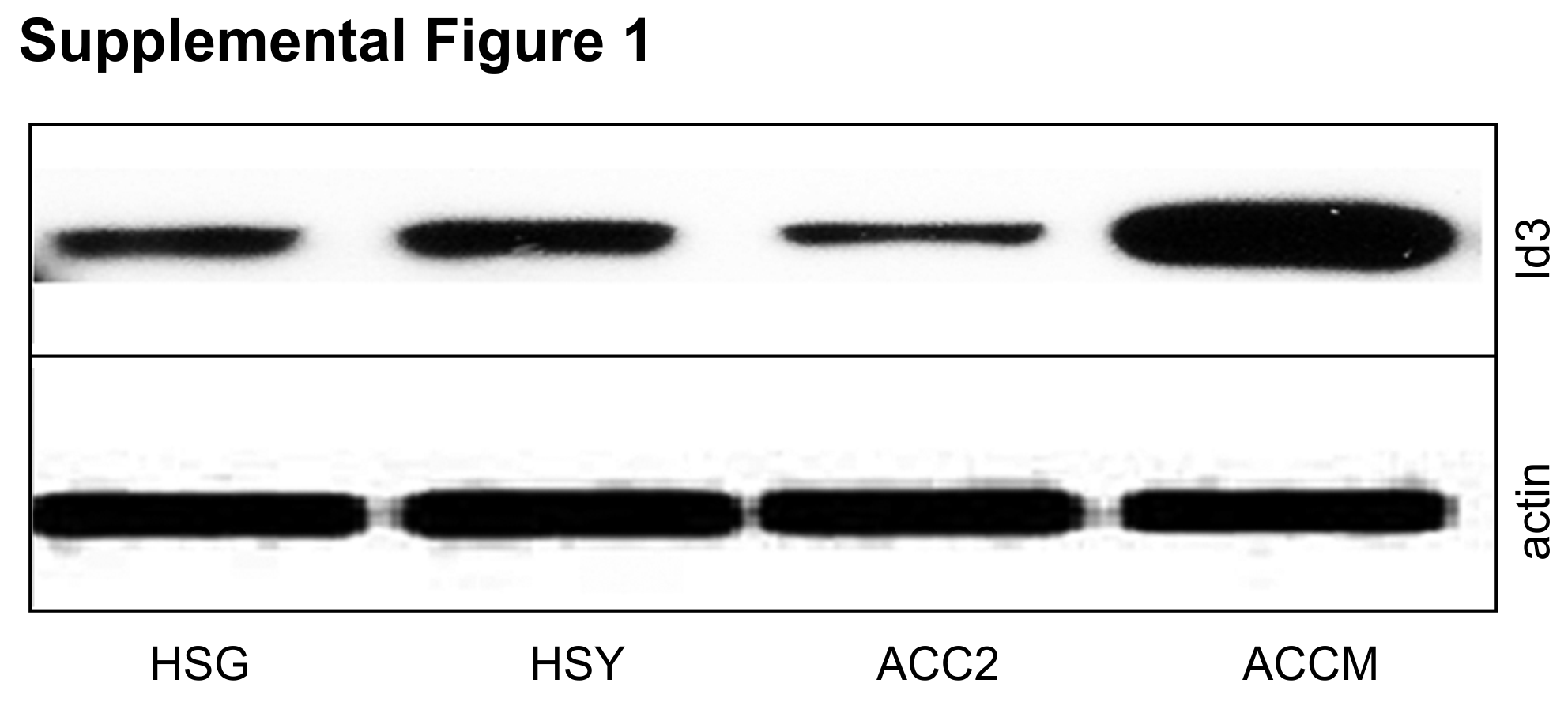

Supplement: Additional file 1: Figure S1 — Id3 protein expression in human SGC cell lines. HSG, HSY, ACC2 and ACCM cells were analyzed for expression of Id3 by Western blotting. Loading control was carried out by stripping the blot and re-probing with an anti-actin antibody. [file 1471-2407-13-141-S1.tiff]

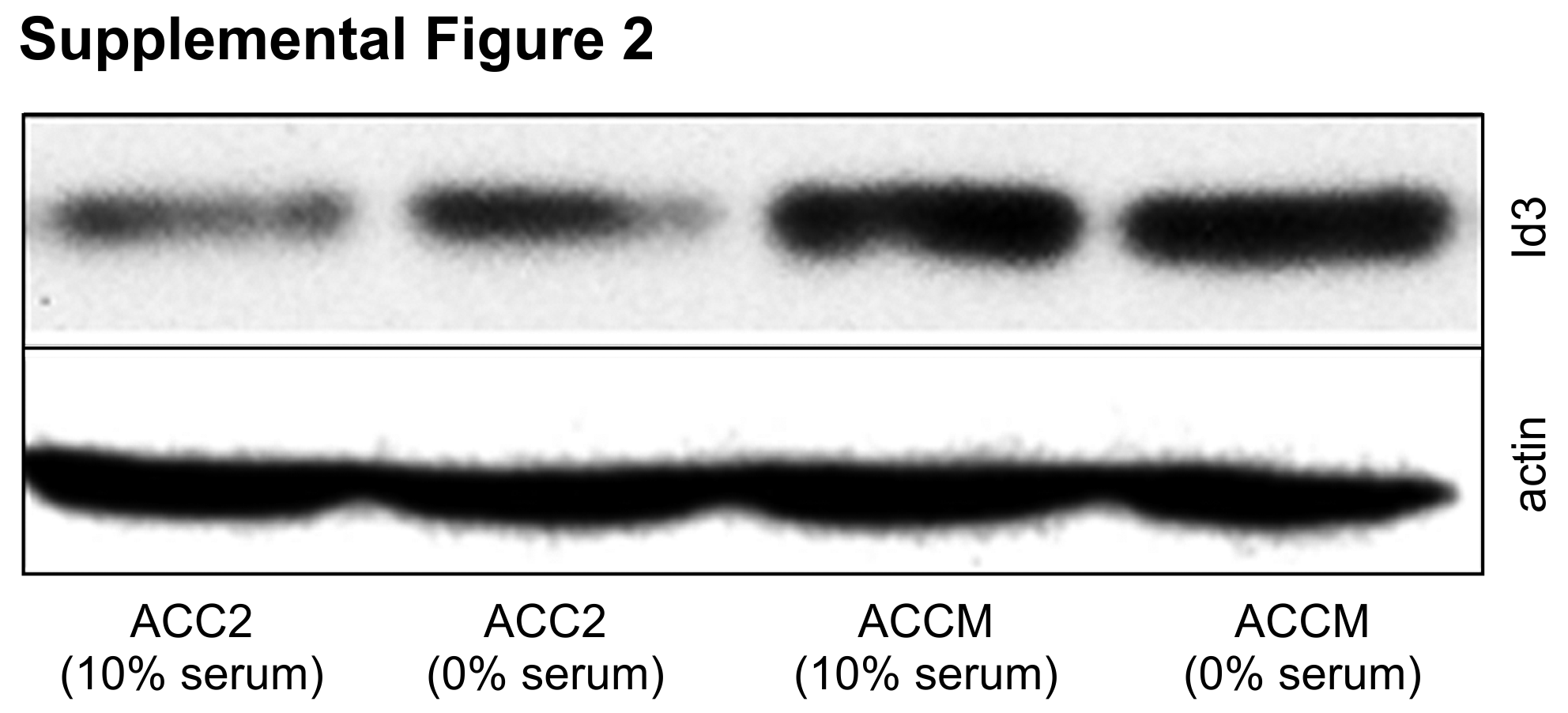

Supplement: Additional file 2: Figure S2 — Id3 protein expression in SGC cells in the presence or absence of serum. Western blot comparing the levels of Id3 protein expression in ACC2 and ACCM cells cultured in 10% FBS or in serum-free medium (0%) is shown. [file 1471-2407-13-141-S2.tiff]
